# Ultra‐Tough Self‐Healing Hydrogel via Hierarchical Energy Associative Dissipation

**DOI:** 10.1002/advs.202303315

**Published:** 2023-07-28

**Authors:** Zhi Zhao, Yurong Li, Haibin Wang, Yupeng Shan, Xuemei Liu, Mengfei Wu, Xinping Zhang, Xiaoyan Song

**Affiliations:** ^1^ Key Laboratory of Advanced Functional Materials Education Ministry of China Faculty of Materials and Manufacturing Beijing University of Technology Beijing 100124 China; ^2^ Institute of Information Photonics Technology Faculty of Science Beijing University of Technology Beijing 100124 China; ^3^ Department of Engineering Mechanics Beijing University of Technology Beijing 100124 China

**Keywords:** associative energy dissipation, hierarchical network, rational design, self‐healing, ultra‐tough hydrogel

## Abstract

Owing to high water content and homogeneous texture, conventional hydrogels hardly reach satisfactory mechanical performance. Tensile‐resistant groups and structural heterogeneity are employed to fabricate tough hydrogels. However, those techniques significantly increase the complexity and cost of material synthesis, and have only limited applicability. Here, it is shown that ultra‐tough hydrogels can be obtained via a unique hierarchical architecture composed of chemically coupled self‐assembly units. The associative energy dissipation among them may be rationally engineered to yield libraries of tough gels with self‐healing capability. Tunable tensile strength, fracture strain, and toughness of up to 19.6 MPa, 20 000%, and 135.7 MJ cm⁻^3^ are achieved, all of which exceed the best known records. The results demonstrate a universal strategy to prepare desired ultra‐tough hydrogels in predictable and controllable manners.

## Graphical abstract

This work explores a novel strategy based on sequential self‐assembly to rationally create ultra‐tough self‐healing hydrogels, which for the first time allows for demand‐oriented precursor design by revealing the relationship between the molecular structure of reactants and the mechanical properties of hydrogels. Libraries of ultra‐tough hydrogels are prepared, whose mechanical properties exceed the best‐known records.

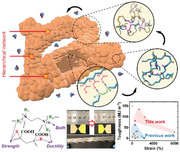

## 1 Introduction

Hydrogels find broad applications in sensing,^[^ ^1^ ^]^ energy storage,^[^ ^2^ ^]^ smart materials,^[^ ^3^ ^]^ and environmental protection.^[^ ^4^ ^]^ The mechanical toughness of hydrogels has great impact on their proper functioning since real devices usually work for extensive hours under load, impact, or structural deformation. Conventionally, hydrogels are hydrophilic polymeric networks that contain a significant portion of water.^[^ ^5^ ^]^ The deficiency of solid materials makes it difficult to form stiff structures. Meanwhile, the homogeneous texture does not support efficient energy dissipation to yield high robustness.^[^ ^6^ ^]^ To address the issues, a number of approaches have been proposed. One popular idea is to incorporate tensile‐resistant units such as micellar crosslinkers,^[^ ^7^, ^8^ ^]^ force responsive groups,^[^ ^9^ ^]^ peptide crosslinkers,^[^ ^10^ ^]^ covalent organic frameworks,^[^ ^11^ ^]^ and ionic crosslinking points^[^ ^12^, ^13^ ^]^ into hydrogel, which absorb energy during mechanical deformation. Alternatively, introducing structural heterogeneity via molecular rearrangement is another feasible solution. Phase separation,^[^ ^14^, ^15^ ^]^ partial polymer crystallization^[^ ^16^, ^17^ ^]^ and polymer alignment^[^ ^18^, ^19^ ^]^ could take place upon thermal annealing, freezing‐thawing or geometric constraint, leading to heterogeneous texture in hydrogel. The formation of alternating stiff and elastic domains enhances hydrogel's comprehensive performance.^[^ ^12^, ^20^ ^]^ By employing multiple strengthening methods, it is possible to further boost the mechanical performance of hydrogels.^[^ ^21^, ^22^ ^]^

Nevertheless, many limitations still exist for current methods. The development of tensile‐resistant groups usually requires extensive organic synthesis, which causes increased experimental complexity and cost. Post‐fabrication treatment is a necessity for the rearrangement of polymers. Not only does it consume additional time, but it also has compatibility issues. Bulky, mechanically unstable and irregularly shaped objects, for example, would be very difficult to treat properly. Due to the irreversible nature of many force responsive groups and treating processes, a great portion of existing ultra‐tough hydrogels do not possess self‐healing abilities, which has negative impact on their working lifetime and long‐term mechanical stability. Last but not least, previous examples were all developed on a try‐and‐error base. There lacks a universal strategy to rationally design super robust hydrogels in a demand oriented fashion.

Here we devised an innovative strengthening strategy for hydrogels based on engineering the spatial organization and collective behavior of coupled self‐assemblies. By mixing hydrogen donor and acceptor with specific chemical features together, unique hierarchical architectures could be spontaneously established. Sequentially formed multiple levels of functional units tightly interlocked within the architecture, which were able to withstand extreme stress and strain via hierarchical energy associative dissipation (HEAD). Tensile strength, fracture strain and toughness of up to 19.6 MPa, 20 000% and 135.7 MJ cm^−3^ have been achieved, all of which exceed the best known records. The mechanical performance of HEAD gels strongly correlated with the structure of reactants, which for the first time allowed for a demand‐oriented rational design of gel precursors. The self‐healing ability, as well as underwater stability of HEAD gel was also investigated to demonstrate their superior robustness.

## 2 Results and Discussion

### 2.1 The Formation and Characteristics of HEAD Gels

**Figure 1:**
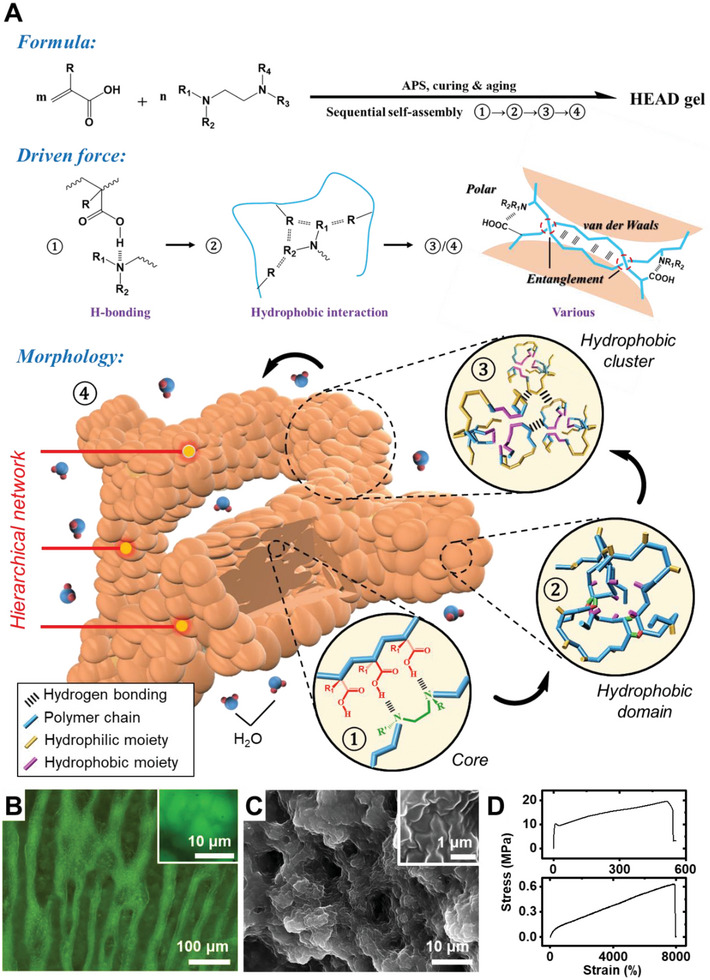
Formation of HEAD gels. A) The formation formula, mechanism and characteristic structure of HEAD gels. B) A representative microscopic image of HEAD gels. Inset: a magnified image of a single thread. C) A representative SEM image of HEAD gels. Inset: a magnified area of C. D) Representative tensile curves showing the extraordinary property of a couple of HEAD gels.

The experimental procedure for preparing HEAD gels was very simple, involving only a mixing of all the reactants following preset recipes (Table S1, Supporting Information). Driven by synergically worked intermolecular assemblies, a hierarchical polymer network would spontaneously form when hydrogen donor and acceptor possessing specific chemical features were polymerized together under suitable concentrations (**Figure** **1**A). This one‐pot fabrication of ultra‐tough hydrogel is distinctive from all previous ones as it does not rely on any special synthetic routes or post‐fabrication treatment. Interestingly, HEAD gels could be made from very common reactants, such as acrylic acid (AAc) and N,N,N′,N′‐tetramethylethylenediamine (TEMED). A wide variety of other species were also applicable, whose names and structures were summarized in Figure S1 (Supporting Information).

A HEAD gel typically involved four sequential levels of self‐assembly (Figure 1A), whose formation and characters were investigated in detail in latter sections. Hydrogen bonding was known to be the very first interaction formed between macromolecules containing hydrogen donors and acceptors.^[^ ^21^, ^22^ ^]^ Here carboxyl groups on the polymer chain were hydrogen donors and amine groups were hydrogen acceptors. As multidentate hydrogen bonds were established, adjacent polymer chains could be effectively grouped, yielding a “core” that functioned as a physical crosslinker. Hydrophobic moieties brought within an appropriate distance could associate with each other, forming hydrophobic domains (e.g., micro‐granules) and making the gel opaque.^[^ ^14^, ^15^ ^]^ Multiple hydrophobic domains might aggregate into bigger clusters via hydrogen bonding/columbic interaction between polar groups, van der Waals forces among non‐polar segments and entanglement of polymers, all of which had been extensively studied in previous researches.^[^ ^22c‐e^ ^]^ Eventually, many clusters got connected and yield a hierarchical network.

The existence of structural heterogeneity was revealed under fluorescent microscopy (Figure 1B). Network patterns were found existing in HEAD gels, whose threads were composed of clusters of tiny grains. Compared with surrounding area, the network was much brighter, indicating a higher density in organics. Such a discovery was verified by SEM (Figure 1C). A number of nano‐sized granules and their clusters had been observed, which corresponded to the hydrophobic domains and hydrophobic clusters.

With the presence of hierarchical network, HEAD gel was able to achieve superior toughness. First, mutually interlocked self‐assemblies made the hierarchical network hard to break so the rupture of hydrogel demands greater force and energy. In addition, the non‐covalent intermolecular interactions (e.g., hydrogen bonding, hydrophobic interactions) were highly reversible in nature, which might contribute to the toughness through two major pathways. First, reversible bonds could undergo many broken‐regeneration cycles during slow mechanical deformations, which repeatedly consumed energy. Moreover, the dynamic regeneration of intermolecular bonds was critical for hydrogels to withstand repeating loads, and made the hydrogels mechanically stable in real applications. Tensile test (Figure 1D) revealed HEAD gels could achieve extraordinary tensile strength (19.6 MPa) and elasticity (strain level of ≈8000%). In comparison, conventional homogeneous hydrogels had much poorer properties.^[^ ^23^, ^24^ ^]^

### 2.2 Construct Appropriate Core in HEAD Gels

**Figure 2:**
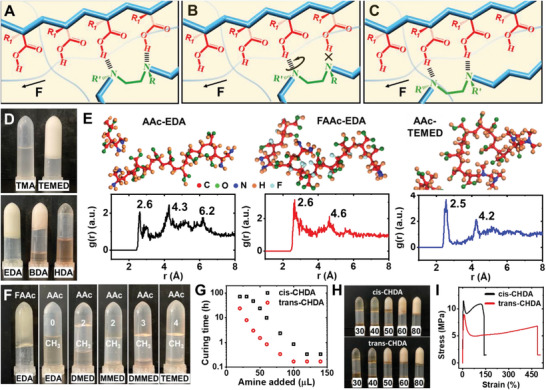
Principles to design z‐core. A–C) Scheme showing the origin of ductility of z‐core. D) Pictures of several precursors after incubation. Top: M1 and M18. Bottom: M4, M13 and M14. E) Snapshots of simulated structure and radial distribution of N‐O distances of three representative systems. F) Pictures of several gel precursors after incubation. From left: F1, A1, A13, A14, A15 and A17. G) Measured curing time as a function of added amine. H) Pictures of cis‐ (M57‐M61) and trans‐CHDA (M62‐M66) gels with different amount (µL) of amine. I) Differences in the tensile behavior of cis‐ (M61) and trans‐CHDA (M66) gel.

To ensure successful preparation of HEAD gels, appropriate core structure, i.e., multidentate hydrogen bonding crosslinked segments, had to be established first. It was found that 1,2‐diamine units were a key for this process (**Figure** **2**A). Since polyacid chains were made from acrylic acid and its derivatives, the carboxyl groups were evenly separated by three carbon atoms. The specific geometry of 1,2‐diamine and polyacid chains allowed them to form a zipper like core (z‐core). When their association was partially destructed, molecular movement could quickly rebuilt the bonding at a neighboring position, leading to sliding, instead of dissociation, between polymers (Figure 2B,C), which greatly promoted the ductility of hydrogel. This working mechanism was similar to that of actual zippers, with carboxyl groups acting as the teeth and 1,2‐diamine as the slider.

The necessity of having 1,2‐diamine unit had been studied (Figure 2D). First, the importance of multidentate bonding was verified. Mixing MAAc and TEMED was able to yield HEAD gels. When TEMED was replaced by TMA, no gel would form. This was because that compared with monodentate amines (TMA), diamine (TEMED) bound polyacid chains at a much higher strength owing to the chelation effect.^[^ ^25^ ^]^ Consequently, the 1,2‐diamine unit served as an effective crosslinker for HEAD gels. Next, the most preferred spatial distribution of amine groups was investigated. It is possible to prepare MAAc based HEAD gels using 0.46 mol L^−1^ EDA. Employing BDA or HDA of the same concentration resulted in incomplete gelation. The results demonstrated 1,2‐diamine led to the most stable z‐core. The greater binding affinity of EDA (1,2‐diamine) to polyacids over BDA and HDA (1,4‐ and 1,6‐diamine) had been attributed to the chelation ring size effect.^[^ ^26^, ^27^ ^]^ With the increase in size of chelation ring, the relatively fixed binding geometry became more and more entropically unfavorable. Additional tests demonstrating the preference of 1,2‐diamine were included in Figure S2A (Supporting Information). It is worthwhile to note that the diamine unit needed be incorporated into polymers so the z‐core could function as a crosslinker (Figure 1A). This might be readily achieved during radical polymerization (Figure S2B and Supporting Text, Supporting Information).^[^ ^28^, ^29^ ^]^ No gel would form if only free amines were added (Figure S2C, Supporting Information).

The establishment of z‐core was also affected by several other factors. The strength of individual hydrogen bond could be enhanced by adding electron withdrawing groups on hydrogen donors, which greatly facilitated the formation of z‐core. Meanwhile, hydrophobic groups in the vicinity of hydrogen bonds could serve as a shield to repel water and improved the stability of z‐core. MD simulations (Figure 2E) showed EDA bound to polymerized FAAc (2‐fluoroacrylic acid) more tightly than pAAc, as demonstrated by their molecular configurations and the radial distribution g(r) of N‐O distances (r). A similar phenomenon was observed when methyl groups were connected to N atoms. Two well‐resolved g(r) peaks with the shortest r values showed up, indicating the formation of tightly bound, regularly shaped bidentate hydrogen bonding proposed in Figures 1A and **2**A. The first peak corresponded to directly bonded N···H‐O and the second peak belonged to the diagonal N‐O distance (Figure S2D, Supporting Information). Our experimental results well matched simulations (Figure 2F). Under low amine concentrations, FAAc‐EDA and AAc‐TEMED precursor could be cured. In contrast, AAc‐EDA precursor would not solidify. It was also noted that the gelation ability generally increased with the number of methyl groups on N atoms (Figure 2F). Moreover, MMED (N,N'‐dimethyl‐1,2‐ethanediamine) showed a better gelation ability than DMED (N,N‐dimethylethylenediamine), which further confirmed the critical role of multidentate hydrogen bonding in z‐core (Figure 2A). Apparently, adding methyl groups to only one of the amines would not improve binding strength much. When hydrophobic group existed on acidic monomers, it had to attach to the same carbon as the carboxyl group, otherwise an energetically unfavorable configuration would form (Figure S3, Supporting Information).

The conformation of diamine significantly affected reaction dynamics. Trans‐CHDA (cyclohexane‐1,2‐diamine) containing precursors cured at least two times faster than *cis*‐CHDA containing ones (Figure 2G). Apparently, the *trans*‐conformation is dynamically favorable, indicating the most probable configuration of polyacid chains, as well as the characteristic bidentate hydrogen bonding in the core, should all possess a trans‐conformation (Figure 2A). On the other hand, polyacid chains could change their configuration by rotating C─C bonds to fit *cis*‐CHDA. As a result, both reagents were able to yield HEAD gels and the products exhibited similar appearance (Figure 2H). Tensile tests revealed that *trans*‐CHDA containing gels were more flexible while their *cis*‐CHDA containing counterparts were harder (Figure 2I). This was attributed to the reason that *trans*‐diamines enabled faster regeneration of intermolecular self‐assembly. In comparison, *cis*‐diamines tended to induce more intensive polymer rearrangement and led to heavily entangled rigid network.

It is easy to imagine that hydrogen acceptors with multiple diamine units could greatly boost the overall strength of z‐core. For example, when AAc was mixed with EDA, DETA (diethylenetriamine), and PEHA (pentaethylenehexamine), the latter two precursors were able to be cured at an amine concentration of 0.46 mol L^−1^ while the EDA containing one remained uncured (Figure S3C, Supporting Information).

### 2.3 Engineer Phase Separation and Supramolecular Self‐Assembly in HEAD Gels

**Figure 3:**
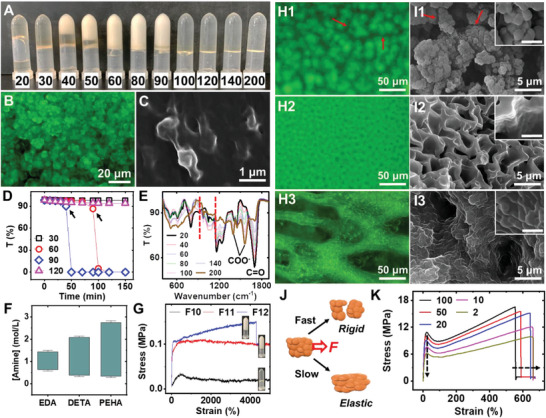
Phase separation and supramolecular self‐assembly in HEAD gels. A) A picture of AAc gels with various amount (µL) of TEMED addition (A16‐A26). B) Fluorescent image of A20 gel (60 in A). C) SEM images of A20 gel (60 in A). D) Timed lapsed transmission of several AAc gels in A. Black arrows indicate the completion of gelation. E) IR spectra of several AAc gels in A. The 960–1124 cm^−1^ region locates between red dashed lines. F) Amine concentrations suitable for phase separation for AAc‐EDA, ‐DETA, and PEHA systems. G) Tensile tests and corresponding pictures of several FAAc hydrogels. H1–H3) Fluorescent images of different advanced structures (M37, M27, and F14). Red arrows in H1 indicate cracks between hydrophobic clusters. I1–I3) SEM images of different advanced structures (M37, M27, and F14). Red arrows in I1 indicate cracks between hydrophobic clusters. Inset: magnified SEM images. Scale bars: 1 µm. J) Scheme showing the dynamic character of advanced structures. K) Tensile behavior of M52 gel as a function of stretching speed (mm min^−1^).

The z‐core alone was weak. However, it shortened the relative distance among polymers thus enabled further hydrophobic interactions and phase separation to generate stronger units. This process was highly dependent on amine concentrations (**Figure** **3**A). At low amine concentrations, no phase separation took place and the hydrogel looked clear (Figure S4A1,B1, Supporting Information). As the amine concentration increased, submicron‐sized hydrophobic domains appeared, which turned the gel into an opaque appearance by light scattering. If the concentration of amine kept growing, hydrophobic domains would eventually disappear (Figure S4A2,B2, Supporting Information). The phase separation was confirmed by both microscopic and SEM characterizations (Figure 3B,C). Fluorescent measurements indicated hydrophobic phases had a higher material density, as revealed by their brighter color. Time‐lapsed evolution of hydrogel structure could be told by spectroscopy (Figure 3D). Precursors with moderate amine concentrations underwent a quick drop in transmission after gelation, suggesting the sequentially formation of z‐cores and hydrophobic domains. In comparison, other systems remained transparent.

The amine‐concentration dependence of phase separation was attributed to the following reasons. When the amine concentration was low, the number of z‐cores was not sufficient to bring the majority of hydrophobic moieties into close contact. Therefore, hydrophobic and hydrophilic groups on the polymer chain randomly aligned in aqueous media (Figure S4C, Supporting Information). As the amine concentration increased, polymeric rearrangement happened spontaneously under the presence of enough z‐cores. Hydrophobic groups would be buried inside while hydrophilic groups would point outwards (Figure S4D), forming hydrophilic shell stabilized hydrophobic domains.^[^ ^30^, ^31^ ^]^ At high amine concentrations, all the hydrophilic carboxyl groups were fixed in the z‐core so the hydrophobic groups had to face outside (Figure S4E, Supporting Information), which was extremely energetically unfavorable. Besides, the pH of precursor rose significantly at high amine concentrations (Figure S5, Supporting Information), leading to ionic COO^−^‐^+^NR_3_ bonding that was prone to hydrolysis (Figure S4E, Supporting Information). As a result, no hydrophobic domains would form. FT‐IR spectra showed when the amine concentration increased, the position of ─CH_2_ and ─CH_3_ stretching bands blue shifted to a greater wavenumber (Figure S4F–G, Supporting Information). This reflected the removal of hydration layer from hydrophobic groups and the formation of hydrophobic interactions.^[^ ^33b,c^ ^]^ Meanwhile, the spectral variation within 960–1124 cm⁻^1^ showed a distinctive trend (Figure 3E), whose magnitude exhibited obvious correlations with the presence of phase separation (Figure 3A). This spectral region mainly contained C─N stretching, N─H rocking, and C─O─H bending bands.^[^ ^32^, ^33^ ^]^ The phenomenon above indicated interactions between carboxyl groups and amine groups (e.g., hydrogen bonding in z‐core) were likely strengthened due to denser molecular packing in hydrophobic domains. Additional discussions on the IR spectra are included in the Supporting Information.

The phase separation was also regulated by other factors. Extending the length of amine would dramatically enhance the tendency of phase separation (Figure 3F). Having electron withdrawing groups or hydrophobic groups on the polyacid chain helped broaden the phase separation window while hydrophobic groups on the amine narrowed it down (Figure S6, Supporting Information). The corresponding mechanism was described in supplementary information. Additional IR tests revealed enhanced bands within 960–1124 cm^−1^ were generally seen when obvious phase separation happened, regardless of the composition of hydrogel (Figure S6, Supporting Information).

The presence of hydrophobic domains significantly toughened hydrogels by its own strength and the strengthening effect on z‐core (Figure 3G). Compared with transparent gels, opaque gels possessed a much higher tensile strength. The mechanical properties of opaque gels, in comparison, did not vary as significantly.

Under proper conditions (Figure S7, Supporting Information), hydrophobic phases might further assemble into clusters and more advanced structures including connected giant agglomerations, uniform porous network and hetero‐porous network (Figure 3H1‐I3). Extensive cracks among adjacent hydrophobic clusters were observed in sample M37 (Figure 3H1,I1), which indicated the bonding in individual hydrophobic clusters was strong, but interactions among cluster might be relatively weak. In sample M27 (Figure 3H2,I2), the fusion of hydrophobic clusters was almost complete so a uniform porous network with smooth walls formed. For sample F14, all the hydrophobic clusters connected into a continuous network but individual hydrophobic domains were still distinguishable. In addition, various pores from trenches of hundreds of micrometers to small sub‐micrometer holes had been observed, making the network more heterogeneous. Additional SEM images of advanced structures were included in Figure S7 (Supporting Information). Regardless of their morphology, advanced structures were all composed of heavily merged hydrophobic domains, which effectively coupled a vast number of non‐covalent interactions.

The existence of advanced structures further enhanced the toughness of HEAD gels (Figure S7, Supporting Information) since their deformation would consume huge energy. Compared with z‐core and hydrophobic domains, advanced structures had a much slower formation dynamics due to the enormous groups participated in self‐assembly (Figure S8, Supporting Information). Consequently, their mechanical behavior showed an obvious dependence on deformation rate (Figure 3J–K). Upon prompt stretching, the advanced structures behaved more like a rigid body. A sharp stress peak showed up at very low strain, followed by a clear drop in stress at moderate strains due to the partial destroy of intermolecular connections. When the stretching speed slowed down, advanced structures became more elastic. The initial stress peak was less significant while the measured fracture strain rose.

### 2.4 Rationally Synergize Energy Dissipations to Design Demanded Products

**Figure 4:**
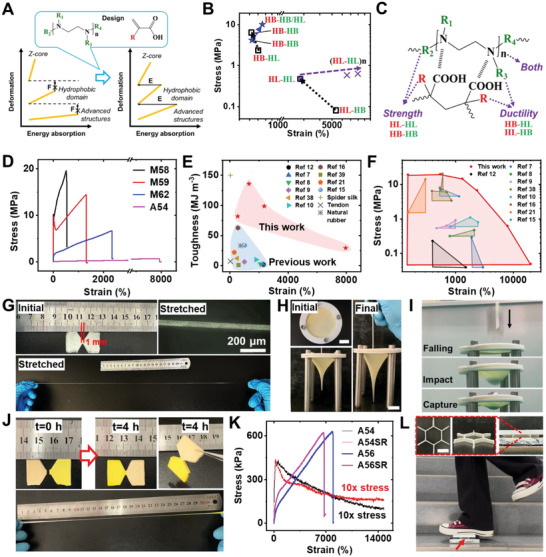
Extraordinary performance of HEAD gels. A) The way to synchronizing energy dissipations. B) The effect of substitutional groups’ property on mechanical performance. Red and green colors indicates substitutional group on acidic monomers and amines, respectively. C) The “molecular structure”‐“mechanical property” relationship in HEAD gels. D) Tensile curves of representative HEAD gels. E) Strain level and toughness of ultra‐tough hydrogels prepared by different methods. F) Tunability in fracture strain and stress (the encompassed area) of ultra‐tough hydrogels prepared by different methods. G) Pictures showing the superior ductility of HEAD gels (A56). H) Pictures of a poking test of A54 gel. Scale bars: 2 cm. I) Pictures of a drop‐ball test of A54 gel. J) The self‐healing of A56 gel. K) Tensile curves of pristine and self‐repaired (SR) HEAD gels. L) Pictures showing a 3.2 g M53 gel scaffold (pointed by the arrow) can support a 50 kg person. Insets: pictures of the scaffold sandwiched between two boards. Scale bar: 2 cm.

Different from previously established toughening methods that focused on only one type of chemical groups or polymer alignments, the HEAD system worked with a collection of energy dissipation units. To create ultra‐tough hydrogels, it is critical to synchronize the effect of z‐core, hydrophobic domains and more advanced structures. The mechanical behavior of HEAD gels could be represented by a spring model (Figure S9, Supporting Information). Ideally, z‐core could slide along polymer chains as long as there was single‐molecular continuity, leading to almost infinite flexibility. However, the strength of z‐core was not satisfactory. Hydrophobic domains and advanced structures were much stronger due to the vast number of intermolecular interactions presented. In contrast, their flexibility was weakened because those characteristic interactions only took effect in localized range.^[^ ^34^, ^35^ ^]^ When an external force was applied, advanced structures began to dissociate at low strain levels and absorbed energy while hydrophobic domains and z‐cores required moderate and high deformation levels to dissociate, respectively. If their mechanical properties differed too much, forces and energies would not be transferred effectively (**Figure** **4**A). For example, hydrophobic domains might not yet started to absorb energy even advanced structures were completely destroyed. To simultaneously yield excellent strength and elasticity, balanced contributions from all components should be introduced via careful molecular design so external stress and its energy could be well dispersed across the entire material.

The substitutional groups on acidic monomers and amines were the key to associative energy dissipation. They can be either hydrophobic (HB), such as –CH_3_, ‐C_2_H_5_, etc. or hydrophilic (HL), e.g. ‐H. A matched hydrophobicity among substitutional groups on hydrogen donors and acceptors resulted in higher strength. According to law of matching water affinity,^[^ ^36^, ^37^ ^]^ such a combination led to stable hydrophobic interactions thus favored hydrophobic domains and advanced structures. Dissimilar hydrophobicity caused reduced strength, but might greatly enhance the elasticity. In this case, the contribution of z‐core became more dominant. For example (Figure 4B), the fracture strain of AAc‐TEMED gel was much greater than AAc‐EDA gel but its ultimate strength was obviously lower (HL‐HB to HL‐HL). A similar trend is observed from MAAc‐EDA gel to MAAc‐TEMED gel (HB‐HL to HB‐HB). The corresponding tensile curves were included in Figure S10A,B (Supporting Information).

To obtained superior comprehensive property, matched and unmatched hydrophobicity should be simultaneously introduced. Employing hydrogen acceptors with alternating hydrophobic and hydrophilic segments was one possible solution (Figure 4B). MAAc‐TEDETA (N,N,N′,N′‐tetraethyldiethylenetriamine) based HEAD gels were found to be much tougher than MAAc‐TEEED (N,N,N′,N′‐tetraethylethylenediamine) gels (HB‐HB/HL vs HB‐HB). Alternatively, using partially substituted amines, such as *cis*‐CHDA and *trans*‐CHDA would also work. In this case, the substitutional group had to be relatively bulky so that the hydrophilic side and hydrophobic side were chemically distinguishable. Details on molecular structures and tensile curves were included in Figure S10C (Supporting Information).

When the hydrophobicity of reactants was fixed, altering the number of 1,2‐diamine unit in amines would significantly influence the comprehensive property. Apparently, this primarily affected the multivalency of hydrogel bonding, which determined the strength and mobility of z‐core. The longer the amine was, the stronger and more stretchable the z‐core became. For hydrophilic reactants, the major contribution of toughness came from hydrogen bonding (z‐core). Therefore, extending the length of amine (HL‐HL to (HL‐HL)_n_) led to increased strength, elasticity, and toughness (Figure 4B and Figure S10D, Supporting Information). For hydrophobic systems, the ultimate toughness was largely determined by hydrophobic interactions. Therefore, the length of amine did not necessarily enhance strength. However, the elasticity of gel could still be improved (Figure S10E, Supporting Information). Of course, simultaneously increasing the concentration of all reactants enhanced mechanical properties. This improvement was not universal, but focused more on tensile strength than elasticity (Figure S10F, Supporting Information).

The clear “molecular structure”–“mechanical property” relationship originated from the unique HEAD architecture (Figure 4C) for the first time enabled demanded‐oriented development of ultra‐tough hydrogels. A flow chart describing how to formulate desired HEAD gels was shown in Figure S11 (Supporting Information). By doing a rational design, libraries of hydrogels with superior comprehensive properties could be readily obtained (Figure 4D and Figure S12, Supporting Information). Combining MAAc and cis‐CHDA yielded extremely hard gels with a tensile strength of 19.6 MPa, strain level of 540% and toughness of 81.8 MJ cm^−3^. Lowing the concentration of cis‐CHDA led to samples with superior comprehensive properties, whose tensile strength, strain level and toughness were 14.5 MPa, 1310%, and 135.7 MJ cm^−3^, respectively. By replacing cis‐CHDA with trans‐CHDA, HEAD gels with balanced hardness and elasticity was fabricated. The tensile strength, fracture strain and toughness were 6.7 MPa, 2360%, and 98.7 MJ cm^−3^. AAc‐PEHA gels exhibited great ductility, whose fracture strain reached 7950%. The corresponding tensile and toughness was 632 kPa and 29.3 MJ cm^−3^.

HEAD gels outperformed the majority of known natural polymers as well as synthetic hydrogels in their comprehensive mechanical performance (Figure 4E).^[^ ^7^, ^8^, ^9^, ^10^, ^12^, ^15^, ^16^, ^21^, ^22^, ^38^, ^39^, ^40^, ^41^ ^]^ For example, HEAD gels could reach a comparable toughness as spider silk, but was 40 times more flexible. Moreover, the mechanical property of HEAD gels could be tuned in a record‐breaking scope (Figure 4F). The tensile strength and fracture stain was able to vary across 0.04–19.6 MPa and 150–20 000%, respectively, which had never been achieved before. Correspondingly, HEAD gels could satisfy almost arbitrary needs on tough hydrogels. Notably, the entire strain‐stress curve of the most stretchable HEAD gels were not able to be recorded completely by available instrument (Figure S12B,C, Supporting Information). Manual tests demonstrated the strain level of those gels exceeded 20 000% (Figure 4G; Video S1, Supporting Information). The final diameter of deformed region was as thin as ∼60 µm, which equaled to a >2000 time shrinkage compared with the original cross‐section (3 mm × 2 mm). The great toughness of HEAD gels might also been demonstrated by poking trials (Figure 4H). As can be seen, a 0.5 mm thick film made by AAc and PEHA could withstand extremely concentrated deformation. Drop‐ball tests (Figure 4I; Video S2, Supporting Information) revealed that a falling ball of 0.82 J kinetic energy could be easily captured by the film.

Another merit of HEAD gel was its self‐healing ability. The hierarchical network was founded on reversible physical interactions, which allowed it to spontaneously recover from damages. As shown in Figure 4J, freshly sliced hydrogel pieces could recombine into continuous material in hours. The bonding strength at interfaces in self‐repaired material was strong enough to withstand extreme stretching (e.g., >20 000%). Tensile test (Figure 4K) revealed that the recovered gel maintained up to 100% of its original strength and toughness. The optimal self‐healing conditions varied among different gels, which were summarized in Table S2 and Figure S13 (Supporting Information). Self‐healing ability is known to be critical to practical applications, which extended the lifetime and stability of hydrogel materials.^[^ ^42^ ^]^ Many previously developed ultra‐tough hydrogels did not possess such a capability due to the application of irreversible chemical crosslinks or phase separation.

Conventional hydrogels severely lost mechanical strength in water due to swelling.^[^ ^43^ ^]^ The unique structure and compositional simplicity of HEAD gels made them highly stable under water. First, the hydrophobic domains were able to protect vulnerable z‐cores from hydrolysis. In addition, HEAD gels did not rely on external chemicals (e.g., salts) to maintain the structural heterogeneity therefore had little risk of material leaching in water. As shown in Figure S12 (Supporting Information), the mechanical toughness of HEAD gels varied little before and after soaking, which was strong enough to support objects that were >16 000 times of their own weight (Figure 4L; Figure S14, Supporting Information).

## 3 Conclusion

In summary, an innovative strategy to construct ultra‐tough hydrogels had been demonstrated. Unlike previously developed counterparts, HEAD gels were founded on collectively worked hierarchical units instead of a single type of chemical group or structure. Experimental investigations confirmed HEAD gels consisted four levels of self‐assemblies, namely the z‐core, the hydrophobic domain, the hydrophobic cluster and the hierarchical network, each of which possessed distinctive morphological features and mechanical behaviors. By properly balancing their contributions, a synergic energy dissipation was achieved, which led to record‐breaking tensile strength, fracture strain and toughness of up to 19.6 MPa, 20 000%, and 135.7 MJ cm^−3^, respectively.

The present method exhibited several advantages over existing fabrication techniques for ultra‐tough hydrogels. First, it only took an adequate mixing of reactants to obtain HEAD gels. No additional synthetic operations or post‐fabrication treatment was required. This impressive simplicity allowed HEAD gels to be prepared at high efficiency, low cost at arbitrary facilities by lightly trained personnel, which was ideal for industrial production. The unique construction of HEAD gels made the reverse engineering of ultra‐tough hydrogels possible. That is, the formulation of precursors could be well deducted from desired mechanical properties via clear rules. This avoided the try‐and‐error based developing protocol and could save tremendous research time. HEAD gels also possessed peerless tunability in mechanical properties and could serve as a universal solution for almost every existing demands in tough hydrogels. Through the combination of self‐healing ability, underwater stability and superior toughness, HEAD gels were extremely robust, which held great promise for practical applications under extreme serving conditions. With the availability of more and more adequate reactants, the HEAD‐gel system will be constantly improved.

## 4 Experimental Section

### Materials

Methacrylic acid (MAAc, 99%), acrylic acid (AAc, 99%), crotonic acid (CAc, 98%), acrylamide (AAm, 99%) and dimethylsulfoxide (DMSO, ≥99.5%) were purchased from Shanghai Macklin Biochemical Co., Ltd. Ethylenediamine (EDA, >98%), 2‐fluoroacrylic acid (FAAc, 98%), N,N,N'‐trimethylethylenediamine (DMMED, 98%), hexamethylenediamine (HDA, 98%), N,N′‐methylenebisacrylamide (Bis, 99%), ammonium persulfate (APS, 99.99%), tetramethylethylenediamine (TEMED) and fluorescein O‐methacrylate (97%) were purchased from Beijing MREDA Technology Co., Ltd. Trimethylamine (TMA, 98%) and N,N'‐dimethyl‐1,2‐ethanediamine (MMED, 98%) were purchased from Energy Chemical. N,N‐dimethylethylenediamine (DMED, 98%) N,N,N′,N′‐tetraethyldiethylenetriamine (TEDETA, ≥97%), 1,4‐diaminobutane (BDA, 98%), pentaethylenehexamine (PEHA, 98%) and diethylenetriamine (DETA, 99%) were purchased from Shanghai Aladdin Bio‐Chem Technology Co., LTD. Cis‐cyclohexane‐1,2‐diamine (cis‐CHDA, 98%) and trans‐cyclohexane‐1,2‐diamine (trans‐CHDA, 98%) were purchased from Bidepharm. N,N,N′,N′‐tetraethylethylenediamine (TEEED, 98%), 1,1,4,7,10,10‐hexamethyltriethylenetetramine (HMTETA, 98+%), N,N,N′,N′‐tetramethyl‐1,3‐propanediamine (TEMPD, ≥98%) and N,N,N′,N″,N‴‐pentamethyldiethylenetriamine (PMDETA, ≥98%) were purchased from Shanghai D&B Laboratory Equipment Co.,Ltd. Methenamine (HMTA, AR) was purchased from Sigma‐Aldrich. Phenylbis(2,4,6‐trimethylbenzoyl)phosphine oxide (Irgacure819, 99+%) was purchased from Beijing Hawk Science & Technology Co., Ltd.

### Sample Preparation

Hydrogel samples were obtained by mixing curable acidic monomers with proper amines. Stock solutions were first prepared by dissolving acidic monomers in deionized water (18.2 MΩ cm^−1^). CAc stock solution needed to be prepared and maintained at 60 °C. To 1 mL of those stock solutions, a variety of amines were added, followed by the addition of 10 wt.% APS solution to initiate in situ polymerization. Detailed recipes of all the precursors were listed in Table S1 (Supporting Information). The typical molar ratio between acidic monomers and amine molecules in a precursor ranged from 2:1 to 20:1. The reaction mixtures were left untouched for 2 days to allow complete cuing and aging. Note the long incubation time was set to ensure a complete gelation of all samples and unify the preparation conditions. Many HEAD gel, e.g., A27‐A48, M24‐M43, etc., could promptly form in hours due to a fast reaction dynamics. For samples used in the mechanical tests, precursors were cast into various molds and cured to form desired shapes. Depending on the recipe, the final water content in HEAD gels ranged from 56 wt.% to 76 wt.%.

To demonstrate the necessity of incorporating 1,2‐diamines into polymers, 3.94 m AAc stock solution was mixed with 10 wt.% APS to conduct polymerization. The precursor was incubated for 7 days before water and TEMED were added. The final composition of the mixture was the same as precursor A20, named as A20′. The mixture was left untouched for another 7 days to see if gelation happened.

### Characterization Methods

Microscopic characterizations were carried out on a Leica DM6 microscope. Hydrogels were molded into a ≈60 µm thin layer prior to characterizations. To improve the visibility of hydrogels under microscope, fluorescein O‐methacrylate was employed to label the samples. Fluorescein O‐methacrylate was first dissolved in DMSO to yield a 50 mg mL^−1^ stock solution. This solution was then mixed with hydrogel precursors at a 1:100 volume ratio. Due to the presence of methacrylate group, fluorescein O‐methacrylate could be incorporated into polymer chains via free radical polymerization following the equation below:


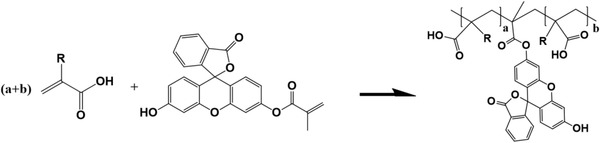


Once the polymers were fluorescent labelled, their structural evolutions could be clearly seen under fluorescent imaging mode. Note the molar ratio between regular monomers and the fluorescent dye was on the order of 3000:1 to 6000:1, so introducing the dye had minimal effect on self‐assembly.

Infrared (IR) spectra of hydrogels were recorded by a FT‐IR spectrometer in the total internal reflection mode (PerkinElmer B420). All samples were sliced into thin layers and completed dried prior to spectral measurements. The dried samples were then crushed into powders and tightly pressed onto the optical window of the spectrometer by an indenter.

The transmission spectra of hydrogels in the UV–vis–NIR regime were recorded by a FLAME‐S‐XR1‐ES spectrometer (Ocean Optics). Precursors of interest were injected into UV cuvette​s with 1 cm path length and characterized in the transmission mode. Time‐lapsed spectra were recorded. Fully cured samples with a maximum transmission below 10% was considered as “having obvious phase separation”.

SEM characterizations were performed with a Nova NanoSEM 200 system. Prior to SEM analysis, all the sample was coated with a ≈10 nm layer of gold by a metal evaporator (Bühler Leybold Optics) to improve the electrical conductivity.

Tensile tests were performed with an Instron 5948 Micro Tester. The stretching speed was set at 20 mm min^−1^ for regular mechanical characterizations, and varied according to experimental purpose in other tests. Drop‐ball tests were performed with an ASR‐2000A drop ball tester. A 55.5 g steel ball was released from 1.5 m above the membrane, whose kinetic energy at impact was 0.82 J.

### Self‐Healing and Underwater Stability Tests

To study the self‐healing ability of hydrogels, samples of interest were cut into pieces by a razor blade, which were then gently pressed together to allow a tight contact. After incubating for a few hours, the self‐healing results were examined by tensile tests. To speed up the self‐healing process, heating at a mild temperature could be applied. Details of the appropriate self‐healing temperature and incubation time were included in Table S2 (Supporting Information).

Underwater stability of HEAD gels was examined by first soaking freshly prepared gel samples in DI water for 24 h. The fully swollen gel was then subjected to tensile tests or other characterizations.

### Molecular Dynamics (MD) Simulations

MD simulations were performed by Material Studio software. The chemical structure of H_2_O and all the reactants are built separately. Molecules being simulated were packed into an amorphous unit cell with a density of 1.0 g cm^−3^ using COMPASS force field. The lattice parameters for each unit cell was *a* = *b* = *c* ≈20 Å, *α* = *β* = *γ* = 90°. Condensed‐phase optimized molecular potentials for atomistic simulation studies (COMPASS force‐field) were applied to calculate the interactions among various atoms.^[^ ^44^ ^]^ The “geometry optimization” function in the “forcite module” was employed to minimize structural energy, in which the convergence tolerance was set at 1 × 10^−4^ kcal mol^−1^ for energy convergence, 5 × 10^−5^ Å for displacement and 0.005 GPa for stress. Geometric optimizations was running automatically to tune the molecular structures until the total energy of the unit cell met the preset parameters. All MD simulations had been investigated by NVE ensembles controlled by Berendsen and Andersen's thermostat. Every cubic cell is analyzed at 298 K for 50 ps.

## Conflict of Interest

The authors declare no conflict of interest.

## Supporting information

Supporting Information

Supporting Information Video 1

Supporting Information Video 2

## Data Availability

The data that support the findings of this study are available from the corresponding author upon reasonable request.
