# Supplementary material for: Ultra‐Tough Self‐Healing Hydrogel via Hierarchical Energy Associative Dissipation
Source: Adv Sci (Weinh). 2023 Jul 28;10(27):2303315. doi: 10.1002/advs.202303315 (PMC10520617; doi:10.1002/advs.202303315)
Supplement: Supplementary file 1 — Supporting Information [file ADVS-10-2303315-s003.pdf]

ADVANCED SCIENCE
Open Access

Supporting Information

for *Adv. Sci.*, DOI 10.1002/advs.202303315

Ultra-Tough Self-Healing Hydrogel via Hierarchical Energy Associative Dissipation

Zhi Zhao, Yurong Li, Haibin Wang, Yupeng Shan, Xuemei Liu, Mengfei Wu, Xinping Zhang and Xiaoyan Song**

Supporting Information

Ultra-tough self-healing hydrogel via hierarchical energy associative dissipation

Zhi Zhao, Yurong Li, Haibin Wang, Yupeng Shan, Xuemei Liu, Mengfei Wu, Xinping Zhang, and Xiaoyan Song**

This file includes:

Supplementary Text
Figure S1 to S14
Table S1 to S2
Captions for Movies S1 to S2

Other Supplementary Materials for this manuscript include the following:

Movies S1 to S2

Supplementary Text

The incorporation of amine groups into polymer chain

Previous investigations showed that 1,2-diamines acted as a secondary initiator under the presence of persulfate (*28*, *29*), which attached to polymer chains during radical polymerization (Figure S2B). The reaction mainly happened at the α-position of N atoms. Under the presence of excess persulfate, the reaction mechanism allowed multiple polymers to be connected to a single diamine. In addition, although TEMED was the predominant secondary initiator being used, other diamines could function in a similar way, as long as their α-position containing C-H moiety. Our experiments demonstrated HEAD gels could be obtained using a number of different diamine species (Table S1).

In-depth analysis of MD simulations

As shown in Figure 2E, two well-resolved g(r) peaks with r equaled to 2.5 Å and 4.2 Å existed in the AAc-TEMED system. Due to the presence of resonance structures, the two O

atoms in carboxyl groups are essentially identical, each can form hydrogen bonding with N (Figure S2D). In MD simulations, each N-O distance was therefore the average distance between N and the two carboxylic O atoms. The first N-O peak in the r-g(r) plot belonged to directly bonded N···H-O. When stable bidentate hydrogen bonding in Figure 2A formed, the two carboxyl groups being involved were fixed diagonally (Figure S2D). In this case, when the first N-O distance was 2.5 Å, the diagonal N-O distance was 4.2 Å. Therefore, the proposed core structure was verified.

Additional discussions on the IR spectra

As the amine concentration increased, the intensity of carboxyl C=O stretching (1698 cm^{-1}) got reduced while the asymmetric (1562 cm^{-1}) and symmetric (1398 cm^{-1}) COO^- stretching became stronger (Figure 3E), suggesting the gradual deprotonation of carboxyl groups due to elevated pH (Figure S5). It was found that the shift of $–CH_2$ and $–CH_3$ bands was the steepest when the volume of added amine changed from 60 μL to 80 μL, indicating a proper amine concentration was the key to trigger the majority of hydrophobic interactions (Figure S4G). When the added amine exceeded 100 μL, the peak position reached a constant value and the corresponding hydrogels appeared to be transparent (Figure S6E). Those observations had verified the scheme in Figure S3E that at high amine concentrations, although hydrophobic interaction still presented, the lack of free hydrophilic groups and the high pH prevented the formation of hydrophobic domains.

The influence of substitutional group on phase separation

As demonstrated by experiments, having electron withdrawing groups or hydrophobic groups on the polyacid chain helped broaden the phase separation window while hydrophobic groups on the amine narrowed it down (Figure S6A-S6C). This discrepancy in the effect of substitutional group was likely caused by the structural character of hydrophobic domains. When hydrophobic domain formed, the z-core acted as a seed to integrate a few hydrophobic segments, rendering a hydrophobic inner sphere. The remaining hydrophilic moieties on the polymer then intertwined the inner sphere to create a hydrophilic shell as a stabilization layer in aqueous media (Figure S4D).

The substitutional group on the polyacid chain enhanced interactions in the inner sphere, therefore reduced the number of required acidic monomers to form a single stable hydrophobic domain. Consequently, phase separation happened across a broader acid:amine range. In contrast, enhancing the hydrophobicity of amines made them stronger seeds so

phase separation could happen at low amine concentrations and each inner sphere contained more hydrophobic segments. This led to a quick consumption of polyacid chains. No sufficient building blocks would be available at high amine concentrations to enable phase separation.

A spring model of HEAD gel

The role of each energy dissipation structure during mechanical deformation could be expressed by a spring model, in which the spring constant $k_1 << k_2 < k_3$ (Figure S9A). The z-core featured the largest elastic range but the smallest spring constant. Hydrophobic domains had moderate elasticity and strength while the advanced structure was strong but rigid. To survive in large stretching, multiple hydrophobic domains and advanced structures had to be tightly connected to share the geometric deformation.

When the system was in lack of hydrophobic interactions, z-cores dominated the mechanical behavior. Although they could withstand large deformation, their low strength made the gel very soft. In contrast, if hydrophobic interaction was too overwhelming, molecular interactions tended to be localized within individual unit so connections among units were lost. In this case, the gel was hard but had very poor ductility. To reach superior comprehensive properties, a chemically balanced network was desired in which the elastic range of united hydrophobic domains and advanced structures was roughly the same as that of z-core (Figure S9B).

Self-repair behavior of various HEAD gels

The optimal self-repair condition varied among different HEAD gels (table S2). In general, softer and elastic gels healed at a faster speed under RT owing to their z-core dominated structure. Tougher gels involving heavy hydrophobic interactions, on the other hand, required a longer healing time at elevated temperatures. For example, A55 and A56 gels could completely recover from damage after a 4 h incubation at RT (Figure 4K). A53 and A54 gels required a higher self-healing temperature (40 °C) and elongated incubation time (Figure S13A). Harder gels such as M47 had very slow self-repair dynamic. To speed up the process, the wound was first transiently heated at about 240 °C for 3 s by a hot plate or iron. The heated area quickly turned clear, indicating the loss of phase separation. The samples were then incubated at 40 °C for 1 day during which new hierarchical structures would form. The as-obtain self-repaired samples possessed partially recovered properties, as shown in Figure S13B.

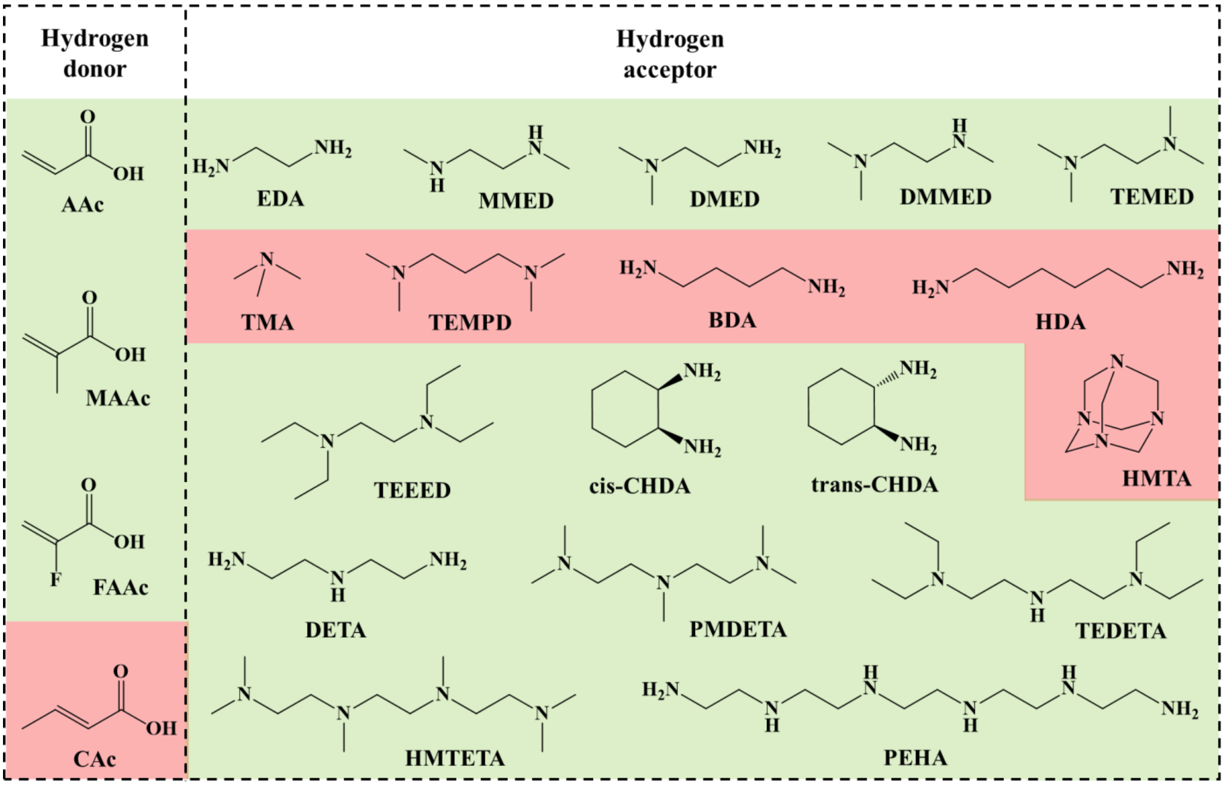

Figure S1. Reactive species tested in the preparation of HEAD gels. The green and red color indicate preferred and undesirable reactants, respectively.

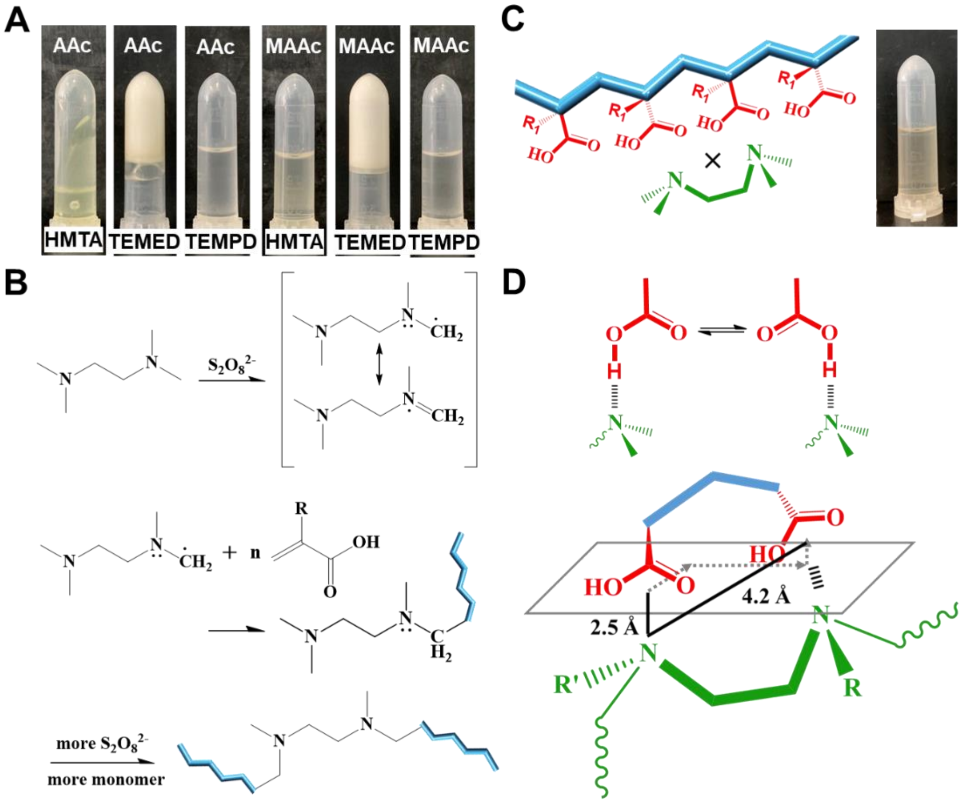

Figure S2. Additional tests on the formation of z-core. (A) Curing tests showing 1,2-diamine (TEMED), rather than 1,1-diamine (HMTA) or 1,3-diamine (TEMPD), was the most preferred structure in HEAD gels. From left: A49, A20, A50, M44, M18, and M45. (B) The reaction mechanism of incorporating diamines into polymer network. (C) Scheme and picture showing free diamines won't cure the gel (A20' in Methods). (D) In-depth analysis on the MD simulation results in Figure 2E.

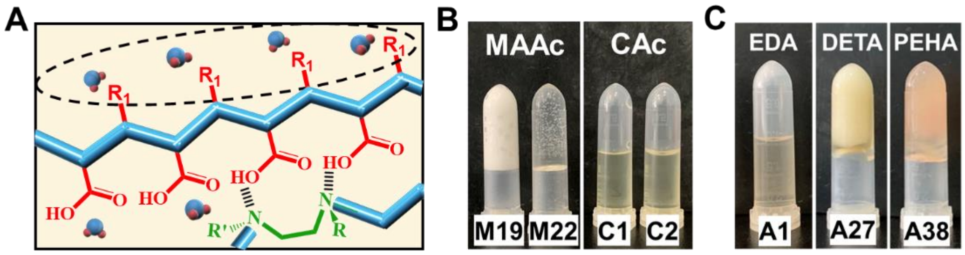

Figure S3. Additional principles to design z-core. (A) Having hydrophobic groups and carboxyl groups on different carbons results in an energetically unfavorable configuration (dash circled region) with regularly aligned hydrophobic groups facing towards aqueous media. (B) Pictures of MAAc and CAc based precursors after incubation, demonstrating the selectivity on position of hydrophobic groups. (C) Pictures reflecting the effect of the number of 1,2-diamine unit on gelation.

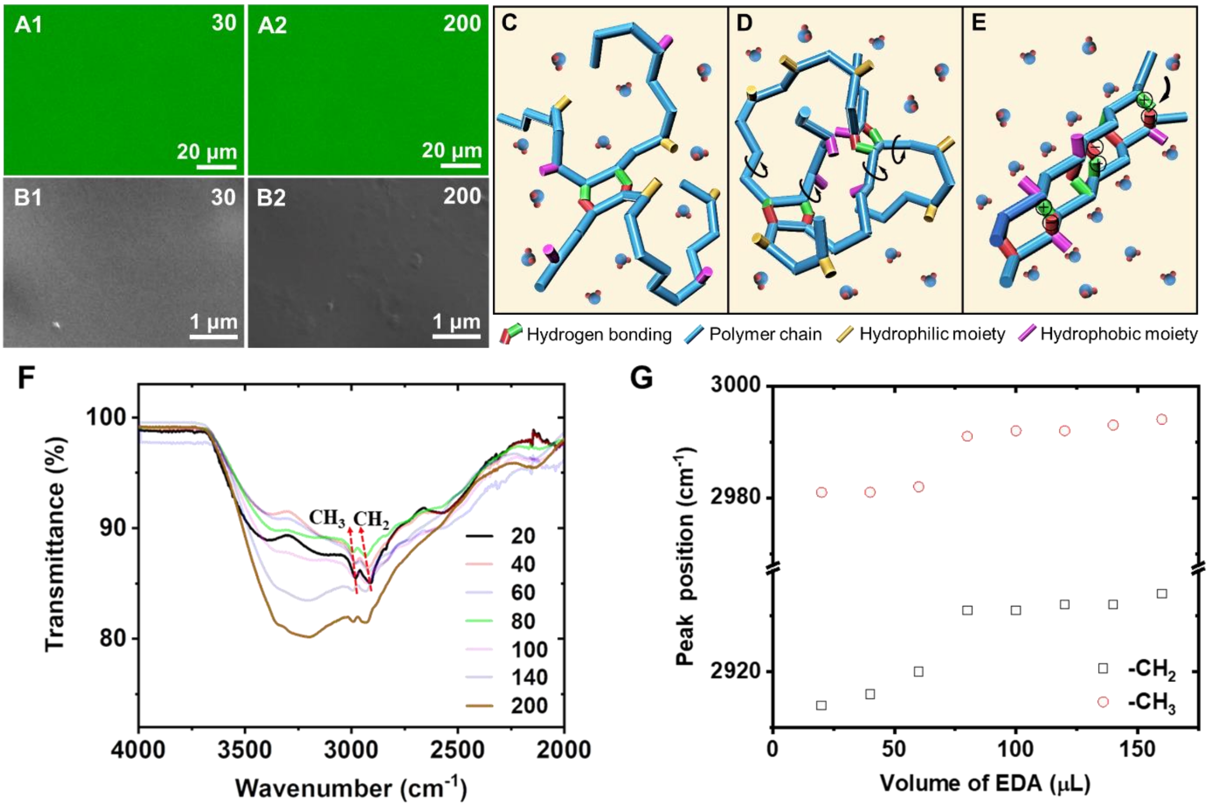

Figure S4. Amine-concentration dependent phase separation. (A1)-(A2) Additional fluorescent image of 30 and 200 gel in Figure 3A. (B1)-(B2) Additional SEM image of 30 and 200 gel in Figure 3A. (C)-(E) Schemes showing the phase separation process at low amine concentration (C), moderate amine concentration (D) and high amine concentration (E). (F) IR spectra of MAAc gels with various amount (μL) of EDA. (G) Peak shift of $–CH_2$ and $–CH_3$ stretching in (F) as a function of added EDA.

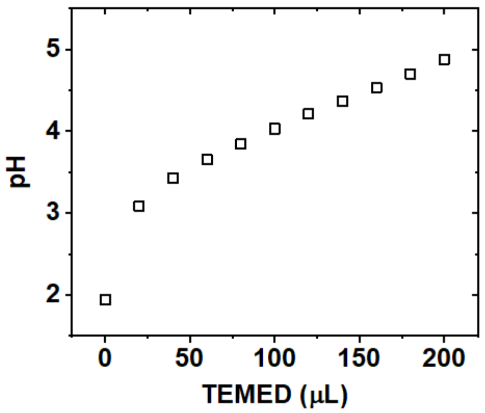

Figure S5. Variation of pH as a function of added TEMED (μL) for a series of AAc-TEMED precursors (A16-A26).

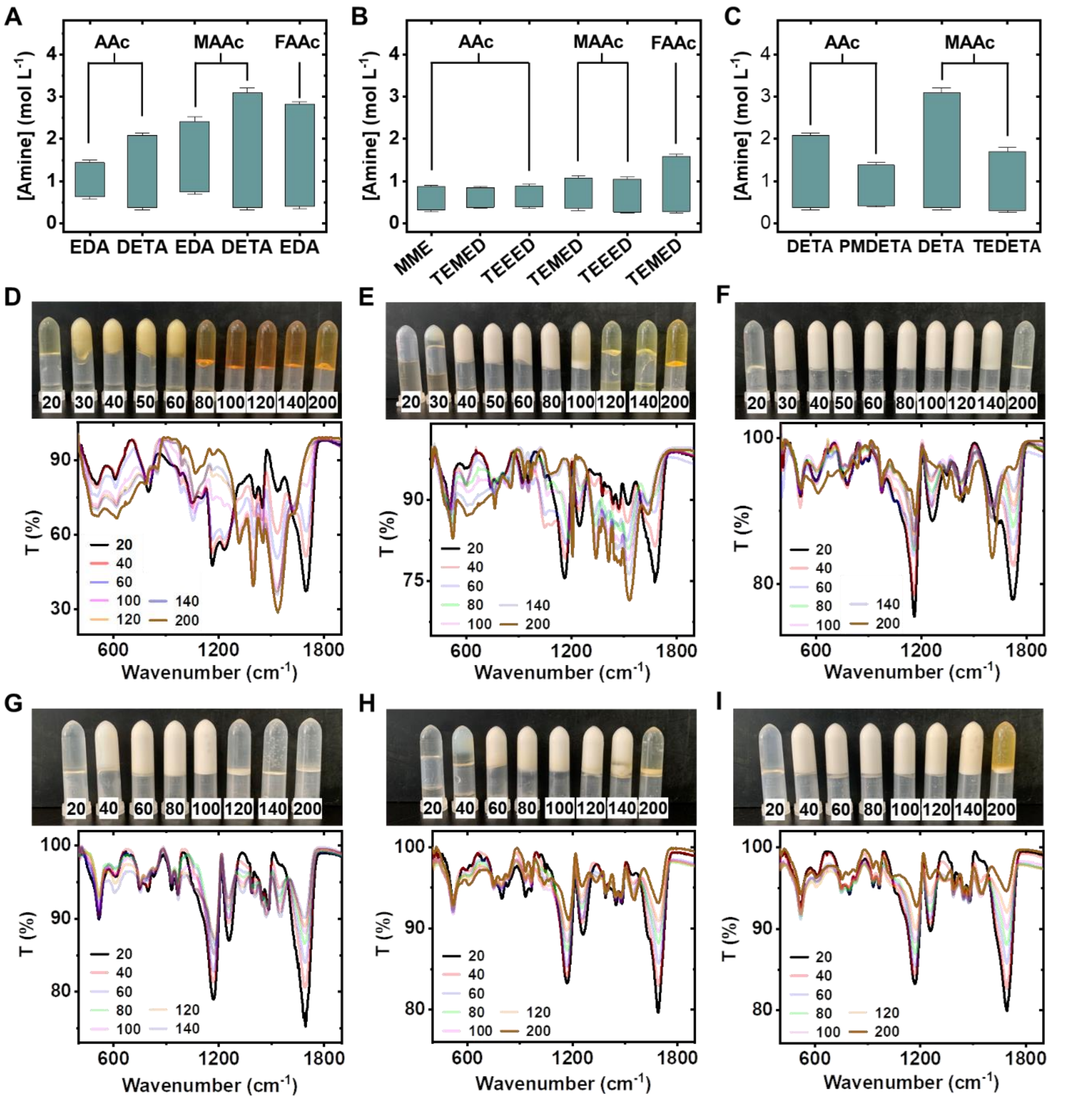

Figure S6. Additional characterizations on phase separation. (A)-(C) Suitable amine concentrations for phase separation (the 'opaque window') in various systems. (D) Picture (from left: A2, A3, A4, A5, A6, A8, A9, A10, A11, A12) and the corresponding IR spectra of AAc gels with various amount (μL) of EDA. (E) Picture (from left: M2, M3, M4, M5, M6, M7, M8, M10, M11, M12) and corresponding IR spectra of MAAc gels with various amount (μL) of EDA. (F) Picture (from left: F10, F11, F12, F13, F14, F15, F16, F17, F18, F20) and corresponding IR spectra of FAAc gels with various amount (μL) of TEMED. (G) Picture (from left: M15, M17, M18, M19, M20, M21, M22, M23) and corresponding IR spectra of MAAc gels with various amount (μL) of TEMED. (H) Picture (from left: M24, M26, M27, M28, M29, M30, M31, M33) and corresponding IR spectra of MAAc gels with various amount (μL) of TEEED. (I) Picture (from left: M34, M36, M37, M38, M39, M40, M41, M43) and corresponding IR spectra of MAAc gels with various amount (μL) of TEDETA.

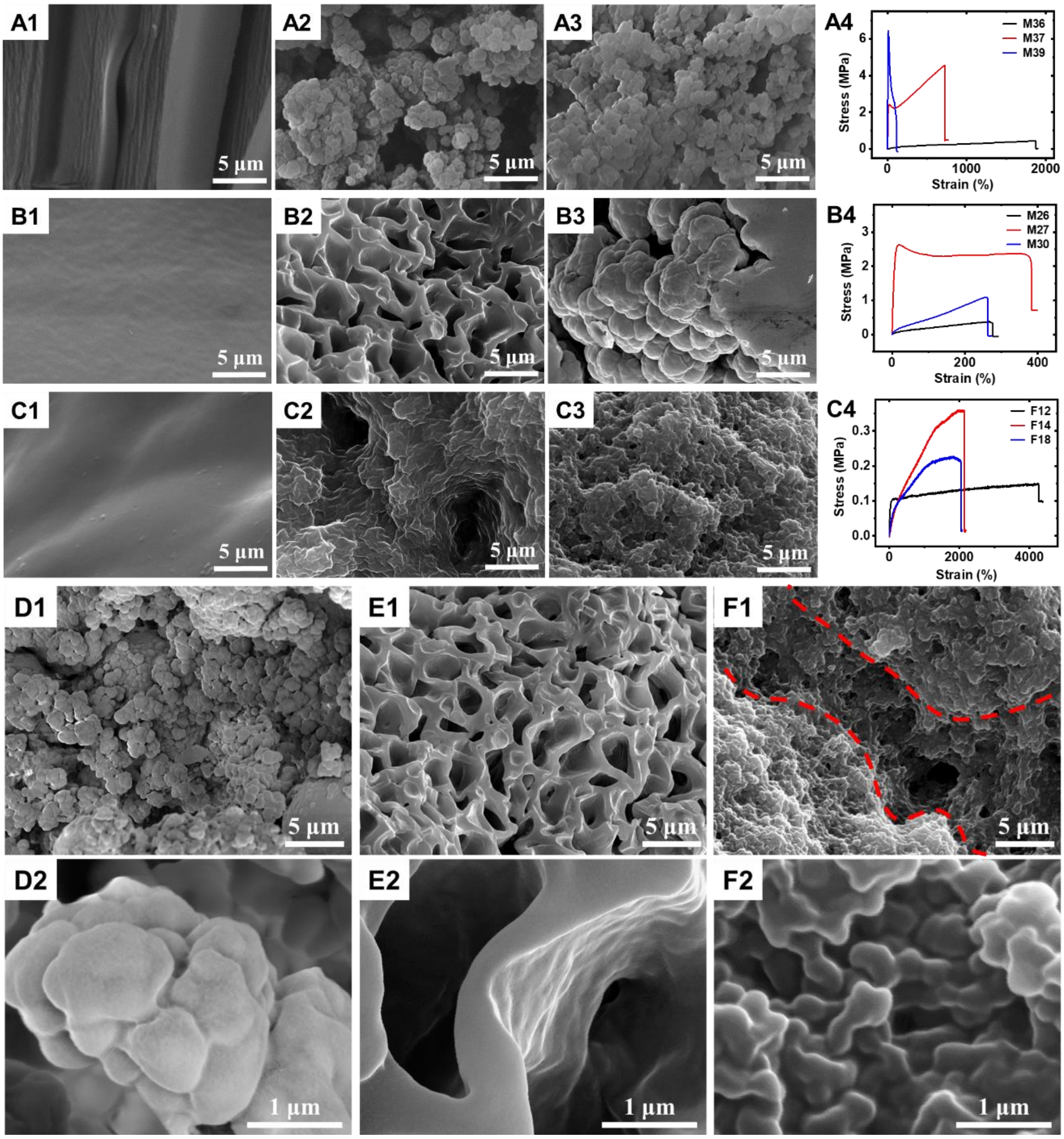

Figure S7. Formation of advanced structures and their influence on mechanical properties. (A1)-(A3) Microscopic morphology of three MAAc-TEBETA gels (M34, M37 and M39). (A4) Tensile curves of MAAc-TEBETA gels. (B1)-(B3) Microscopic morphology of three MAAc-TEEED gels (M24, M27 and M29). (B4) Tensile curves of MAAc-TEEED gels. (C1)-(C3) Microscopic morphology of three FAAc-TEMED gels (F10, F14 and F16). (C4) Tensile curves of FAAc-TEMED gels. (D1-D2) Additional SEM images of M37 gel. (E1-E2) Additional SEM images of M27 gel. (F1-F2) Additional SEM images of F14 gel. Red dashed lines in F1 indicate the trenches found in Figure 3H3.

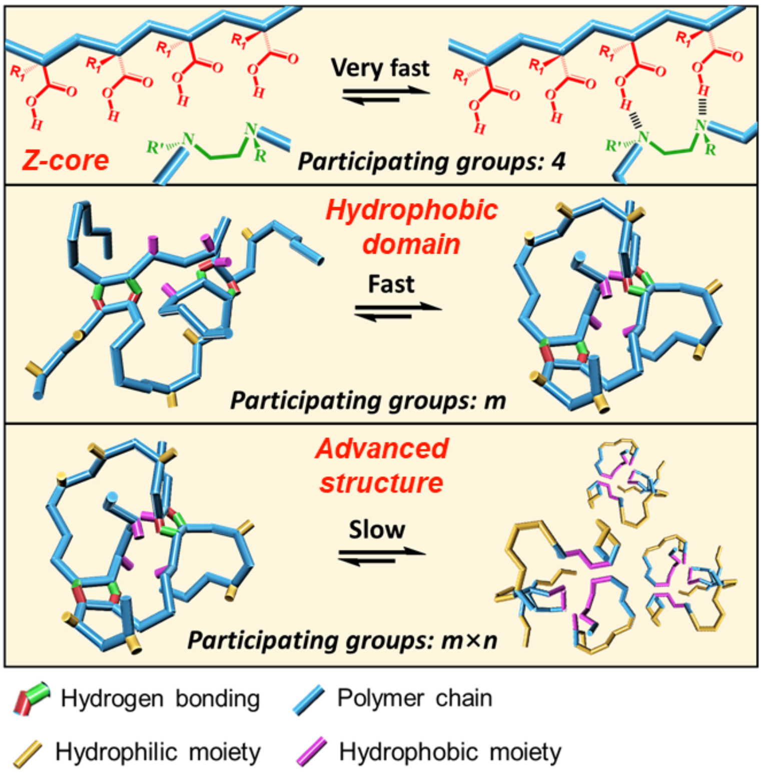

Figure S8. Scheme showing the formation dynamics of different structural components.

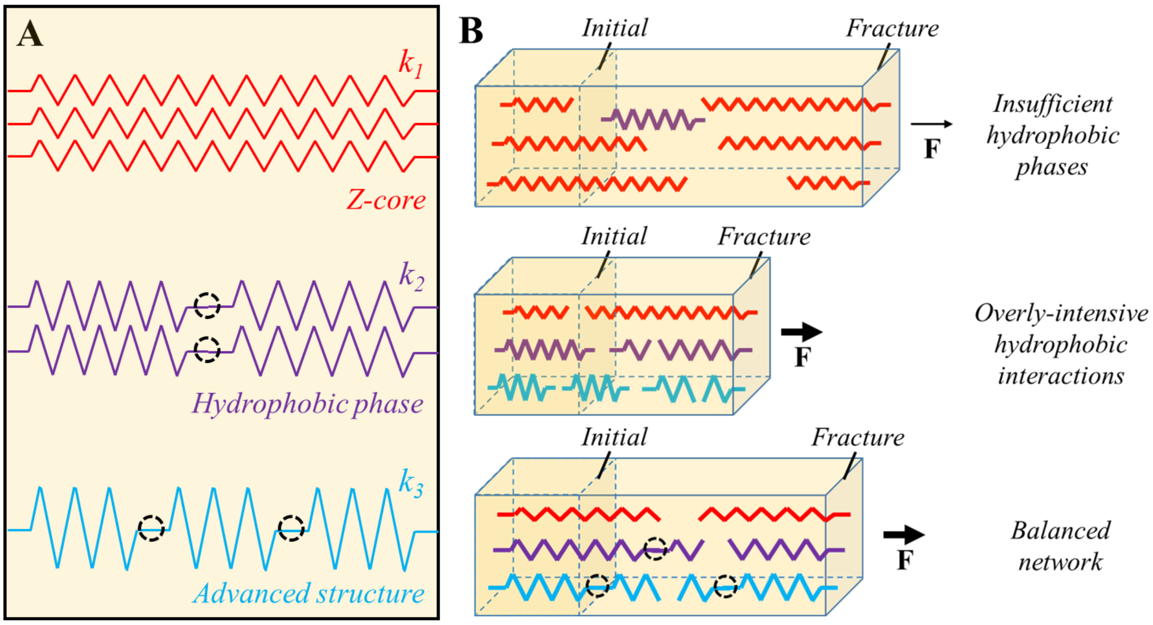

Figure S9. A spring model of HEAD gels. (A) Spring model of each structural component under static condition. Black circles indicate strong connections between different units. (B) Mechanical behavior of various HEAD gels explained by the spring model. Black circles indicate strong connections between different units.

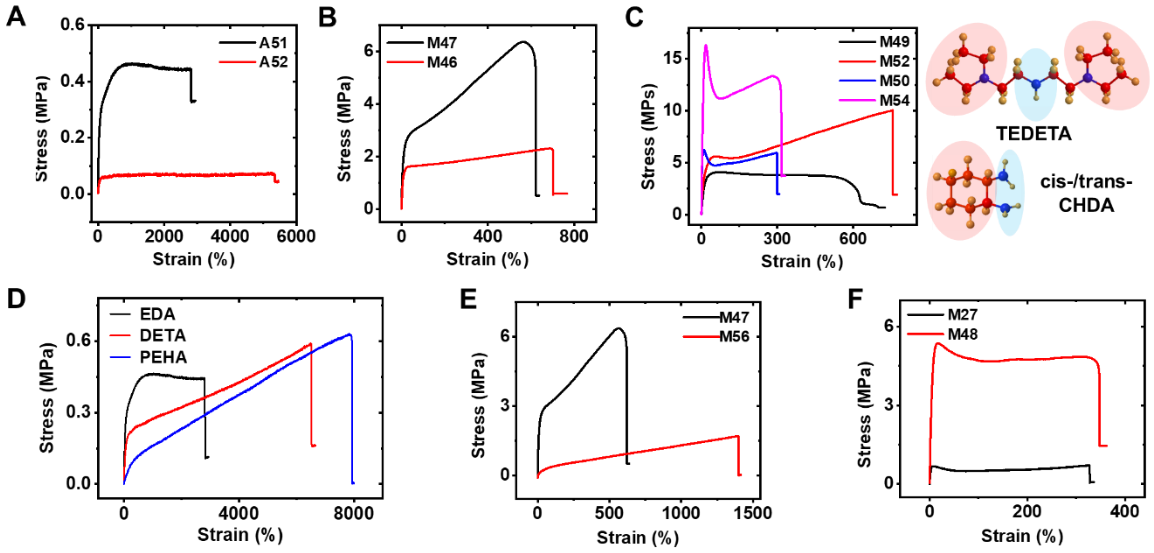

Figure S10. Strategy to tune the property of HEAD gels. (A)-(B) Effect of matching water affinity. (C) Effect of introducing matched and unmatched hydrophobicity. The scheme on the right showing the alternating hydrophobic (red) and hydrophilic (blue) segments in TEDETA and cis-/trans-CHDA. (D) Tensile behavior of HEAD gels as a function of the length of amine (A51, A53 and A54). (E) Longer amines enhanced the elasticity in relatively hydrophobic reaction systems, but showed little correlation with the strength. (F) Effect of concentration of reactants.

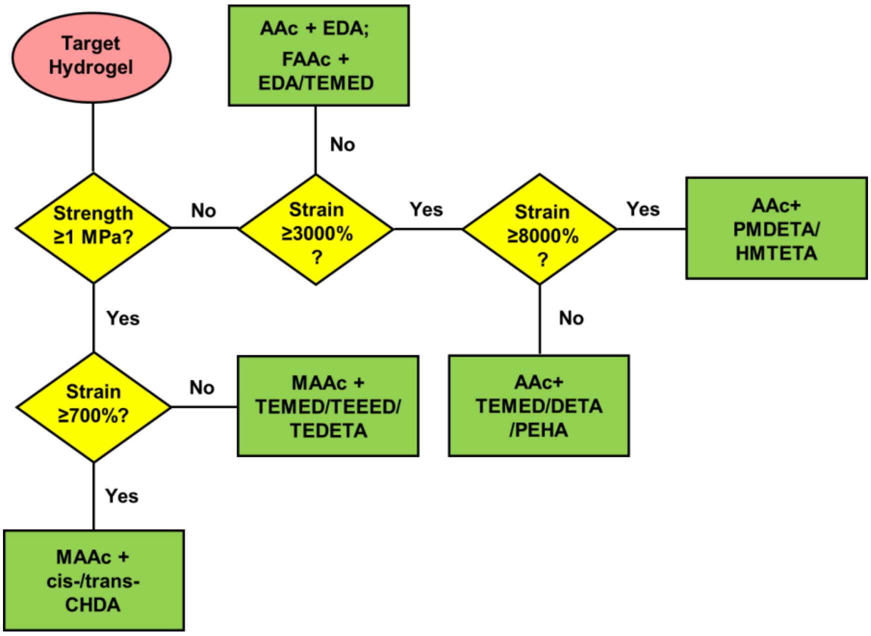

Figure S11. A flow chart describing the design strategy of HEAD gels.

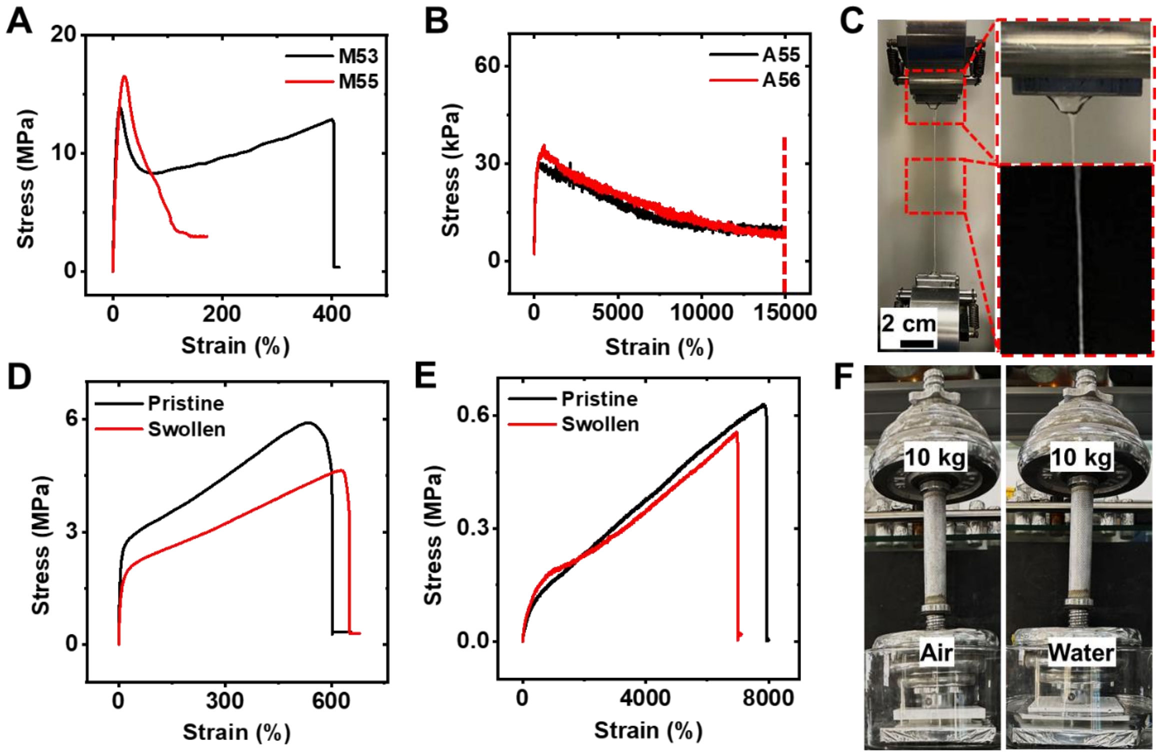

Figure S12. Additional characterizations of HEAD gels. (A)-(B) Additional tensile results. Note the maximum measurable strain was 15000% due to instrumental limit. (C) Pictures of a stretched A56 gel. Material appeared to be sliding out of the shoulder and extended into extremely thin thread, demonstrating the role of z-core. (D) Tensile curves of M47 gel before and after swelling. (E) Tensile curves of A54 gel before and after swelling. (F) Pictures showing the load-bearing capacity of the scaffold in Figure 5L in both air and water.

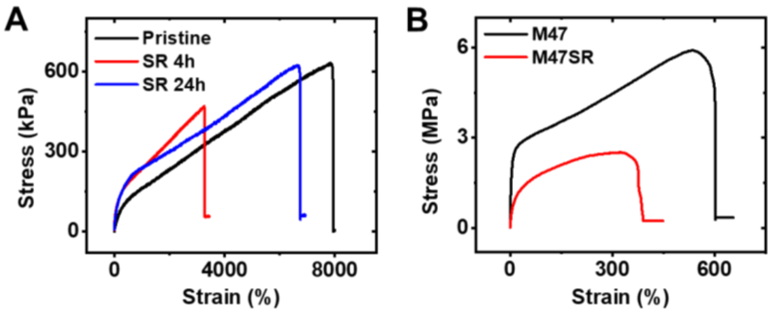

Figure S13. Additional self-healing tests. (A) Tensile test of pristine A56 gel and self-repaired (SR) A56 gels incubated for 4 h and 24 h. (B) Tensile results of pristine and SR M47 gel.

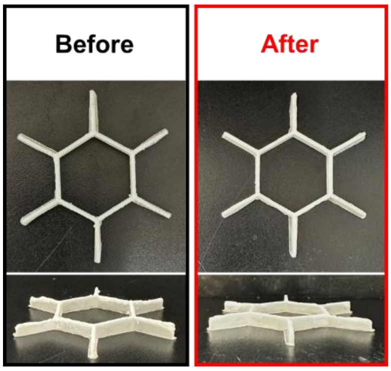

Figure S14. Pictures of the M53 scaffold before and after load tests. No obvious damaged was observed.

Table S1. A list of all the recipes of precursor.

Recipe	Hydrgen donor		Hydrogen acceptor		Donor: acceptor ratio	Water (μL)	10 wt% APS (μL)	Gelation	Forming HEAD gel
	Name	Concentration (M)	Name	Volume (μL)					
A1	AAc	3.94	EDA	13	20.3	187	100	N	N
A2	AAc	3.94	EDA	20	13.2	180	100	Y	N
A3	AAc	3.94	EDA	30	8.8	170	100	Y	Y
A4	AAc	3.94	EDA	40	6.6	160	100	Y	Y
A5	AAc	3.94	EDA	50	5.3	150	100	Y	Y
A6	AAc	3.94	EDA	60	4.4	140	100	Y	Y
A7	AAc	3.94	EDA	65	4.1	135	100	Y	N
A8	AAc	3.94	EDA	80	3.3	120	100	Y	N
A9	AAc	3.94	EDA	100	2.6	100	100	Y	N
A10	AAc	3.94	EDA	120	2.2	80	100	Y	N
A11	AAc	3.94	EDA	140	1.9	60	100	N	N
A12	AAc	3.94	EDA	200	1.3	0	100	N	N
A13	AAc	3.94	DMED	21	20.6	179	100	N	N
A14	AAc	3.94	MMED	21	20.6	179	100	Y	N
A15	AAc	3.94	DMMED	26	19.7	174	100	Y	N
A16	AAc	3.94	TEMED	20	29.5	180	100	Y	N
A17	AAc	3.94	TEMED	30	19.7	170	100	Y	N
A18	AAc	3.94	TEMED	40	14.8	160	100	Y	N
A19	AAc	3.94	TEMED	50	11.8	150	100	Y	Y
A20	AAc	3.94	TEMED	60	9.8	140	100	Y	Y
A21	AAc	3.94	TEMED	80	7.4	120	100	Y	Y
A22	AAc	3.94	TEMED	90	6.6	110	100	Y	Y
A23	AAc	3.94	TEMED	100	5.9	100	100	Y	N
A24	AAc	3.94	TEMED	120	4.9	80	100	Y	N
A25	AAc	3.94	TEMED	140	4.2	60	100	Y	N
A26	AAc	3.94	TEMED	200	3.0	0	100	Y	N
A27	AAc	3.94	DETA	15	28.4	185	100	Y	N
A28	AAc	3.94	DETA	21	20.3	179	100	Y	Y
A29	AAc	3.94	DETA	30	14.2	170	100	Y	Y
A30	AAc	3.94	DETA	40	10.6	160	100	Y	Y
A31	AAc	3.94	DETA	50	8.5	150	100	Y	Y
A32	AAc	3.94	DETA	60	7.1	140	100	Y	Y
A33	AAc	3.94	DETA	80	5.3	120	100	Y	Y
A34	AAc	3.94	DETA	100	4.3	100	100	Y	Y
A35	AAc	3.94	DETA	120	3.5	80	100	Y	N
A36	AAc	3.94	DETA	140	3.0	60	100	Y	N
A37	AAc	3.94	DETA	200	2.1	0	100	N	N
A38	AAc	3.94	PEHA	15	64.3	185	100	Y	N
A39	AAc	3.94	PEHA	25	38.6	175	100	Y	Y
A40	AAc	3.94	PEHA	40	24.1	160	100	Y	Y
A41	AAc	3.94	PEHA	50	19.3	150	100	Y	Y
A42	AAc	3.94	PEHA	60	16.1	140	100	Y	Y

A43	AAc	3.94	PEHA	80	12.0	120	100	Y	Y
A44	AAc	3.94	PEHA	100	9.6	100	100	Y	Y
A45	AAc	3.94	PEHA	120	8.0	80	100	Y	Y
A46	AAc	3.94	PEHA	140	6.9	60	100	Y	Y
A47	AAc	3.94	PEHA	160	6.0	40	100	Y	N
A48	AAc	3.94	PEHA	200	4.8	0	100	N	N
A49	AAc	3.94	HMTA	21	51.0	179	100	N	N
A50	AAc	3.94	TEMPD	67	9.8	133	100	N	N
A51	AAc	6.1	EDA	60	6.8	140	100	Y	Y
A52	AAc	6.1	TEMED	80	11.4	120	100	Y	Y
A53	AAc	6.1	DETA	60	11.0	140	100	Y	Y
A54	AAc	6.1	PEHA	80	18.7	120	100	Y	Y
A55	AAc	6.1	PMDETA	70	18.2	130	100	Y	Y
A56	AAc	6.1	PMDETA	80	15.9	120	100	Y	Y
M1	MAAc	3.94	TMA	70	5.0	130	100	N	N
M2	MAAc	3.94	EDA	20	13.2	180	100	N	N
M3	MAAc	3.94	EDA	30	8.8	170	100	Y	N
M4	MAAc	3.94	EDA	40	6.6	160	100	Y	Y
M5	MAAc	3.94	EDA	50	5.3	150	100	Y	Y
M6	MAAc	3.94	EDA	60	4.4	140	100	Y	Y
M7	MAAc	3.94	EDA	80	3.3	120	100	Y	Y
M8	MAAc	3.94	EDA	100	2.6	100	100	Y	Y
M9	MAAc	3.94	EDA	110	2.4	90	100	Y	N
M10	MAAc	3.94	EDA	120	2.2	80	100	Y	N
M11	MAAc	3.94	EDA	140	1.9	60	100	Y	N
M12	MAAc	3.94	EDA	200	1.3	0	100	N	N
M13	MAAc	3.94	BDA	60	6.6	140	100	Y	Y
M14	MAAc	3.94	HDA	78	6.6	122	100	N	N
M15	MAAc	3.94	TEMED	20	29.5	180	100	Y	N
M16	MAAc	3.94	TEMED	30	19.7	170	100	Y	N
M17	MAAc	3.94	TEMED	40	14.8	160	100	Y	Y
M18	MAAc	3.94	TEMED	60	9.8	140	100	Y	Y
M19	MAAc	3.94	TEMED	80	7.4	120	100	Y	Y
M20	MAAc	3.94	TEMED	100	5.9	100	100	Y	Y
M21	MAAc	3.94	TEMED	120	4.9	80	100	Y	N
M22	MAAc	3.94	TEMED	140	4.2	60	100	Y	N
M23	MAAc	3.94	TEMED	200	3.0	0	100	Y	N
M24	MAAc	3.94	TEEED	20	42.5	180	100	Y	N
M25	MAAc	3.94	TEEED	30	28.3	170	100	Y	N
M26	MAAc	3.94	TEEED	40	21.2	160	100	Y	Y
M27	MAAc	3.94	TEEED	60	14.2	140	100	Y	Y
M28	MAAc	3.94	TEEED	80	10.6	120	100	Y	Y
M29	MAAc	3.94	TEEED	100	8.5	100	100	Y	Y
M30	MAAc	3.94	TEEED	120	7.1	80	100	Y	Y
M31	MAAc	3.94	TEEED	140	6.1	60	100	Y	Y
M32	MAAc	3.94	TEEED	160	5.3	40	100	Y	N
M33	MAAc	3.94	TEEED	200	4.2	0	100	Y	N
M34	MAAc	3.94	TEDETA	20	50.7	180	100	Y	N

M35	MAAc	3.94	TEDETA	30	33.8	170	100	Y	N
M36	MAAc	3.94	TEDETA	40	25.3	160	100	Y	Y
M37	MAAc	3.94	TEDETA	60	16.9	140	100	Y	Y
M38	MAAc	3.94	TEDETA	80	12.7	120	100	Y	Y
M39	MAAc	3.94	TEDETA	100	10.1	100	100	Y	Y
M40	MAAc	3.94	TEDETA	120	8.4	80	100	Y	Y
M41	MAAc	3.94	TEDETA	140	7.2	60	100	Y	Y
M42	MAAc	3.94	TEDETA	160	6.3	40	100	Y	Y
M43	MAAc	3.94	TEDETA	200	5.1	0	100	Y	N
M44	MAAc	3.94	HMTA	21	51.0	179	100	N	N
M45	MAAc	3.94	TEMPD	67	9.8	133	100	N	N
M46	MAAc	6.1	EDA	80	5.1	120	100	Y	Y
M47	MAAc	6.1	TEMED	80	11.4	120	100	Y	Y
M48	MAAc	6.1	TEEED	90	14.6	110	100	Y	Y
M49	MAAc	6.1	TEEED	114	11.5	86	100	Y	Y
M50	MAAc	6.1	TEEED	140	9.4	60	100	Y	Y
M51	MAAc	6.1	TEDETA	80	19.6	120	100	Y	Y
M52	MAAc	6.1	TEDETA	90	17.4	110	100	Y	Y
M53	MAAc	6.1	TEDETA	100	15.7	100	100	Y	Y
M54	MAAc	6.1	TEDETA	110	14.3	90	100	Y	Y
M55	MAAc	6.1	TEDETA	120	13.1	80	100	Y	Y
M56	MAAc	6.1	HMTETA	73	22.7	127	100	Y	Y
M57	MAAc	6.1	cis-CHDA	30	24.4	170	100	Y	N
M58	MAAc	6.1	cis-CHDA	40	18.3	160	100	Y	N
M59	MAAc	6.1	cis-CHDA	50	14.6	150	100	Y	Y
M60	MAAc	6.1	cis-CHDA	60	12.2	140	100	Y	Y
M61	MAAc	6.1	cis-CHDA	80	9.1	120	100	Y	Y
M62	MAAc	6.1	trans-CHDA	30	24.4	170	100	Y	N
M63	MAAc	6.1	trans-CHDA	40	18.3	160	100	Y	N
M64	MAAc	6.1	trans-CHDA	50	14.6	150	100	Y	Y
M65	MAAc	6.1	trans-CHDA	60	12.2	140	100	Y	Y
M66	MAAc	6.1	trans-CHDA	80	9.1	120	100	Y	Y
F1	FAAc	3.94	EDA	13	20.3	187	100	Y	N
F2	FAAc	3.94	EDA	20	13.2	180	100	Y	Y
F3	FAAc	3.94	EDA	30	8.8	170	100	Y	Y
F4	FAAc	3.94	EDA	40	6.6	160	100	Y	Y
F5	FAAc	3.94	EDA	60	4.4	140	100	Y	Y
F6	FAAc	3.94	EDA	80	3.3	120	100	Y	Y
F7	FAAc	3.94	EDA	100	2.6	100	100	Y	Y
F8	FAAc	3.94	EDA	120	2.2	80	100	Y	Y
F9	FAAc	3.94	EDA	150	1.8	50	100	Y	N
F10	FAAc	3.94	TEMED	20	29.5	180	100	Y	N
F11	FAAc	3.94	TEMED	30	19.7	170	100	Y	Y
F12	FAAc	3.94	TEMED	40	14.8	160	100	Y	Y
F13	FAAc	3.94	TEMED	50	11.8	150	100	Y	Y
F14	FAAc	3.94	TEMED	60	9.8	140	100	Y	Y
F15	FAAc	3.94	TEMED	80	7.4	120	100	Y	Y
F16	FAAc	3.94	TEMED	100	5.9	100	100	Y	Y

F17	FAAc	3.94	TEMED	120	4.9	80	100	Y	Y
F18	FAAc	3.94	TEMED	140	4.2	60	100	Y	Y
F19	FAAc	3.94	TEMED	170	3.5	30	100	Y	N
F20	FAAc	3.94	TEMED	200	3.0	0	100	Y	N
C1	CAc	3.94	TEMED	80	7.4	120	100	N	N
C2	CAc	3.94	TEMED	140	4.2	60	100	N	N

Table S2. Summary on self-healing conditions.

Recipe No.	Optimal temperature (°C)	Time	Recovery of toughness
A55	RT	4 h	~100%
A56	RT	4 h	~100%
A53	40	24 h	90%±9%
A54	40	24 h	90%±9%
M47	240	3 s	35%±5%
	40	24 h	

Movie S1.

An A56 gel being manually stretched.

Movie S2.

A drop-ball test.
